# Supplementary material for: Multi-omics protein signaling networks identify sex-specific therapeutic candidates in lung adenocarcinoma
Source: Biol Sex Differ. 2025 Sep 29;16:71. doi: 10.1186/s13293-025-00752-1 (PMC12482505; doi:10.1186/s13293-025-00752-1)

Figure S1. Representative KEGG pathways enriched in the protein signaling network.

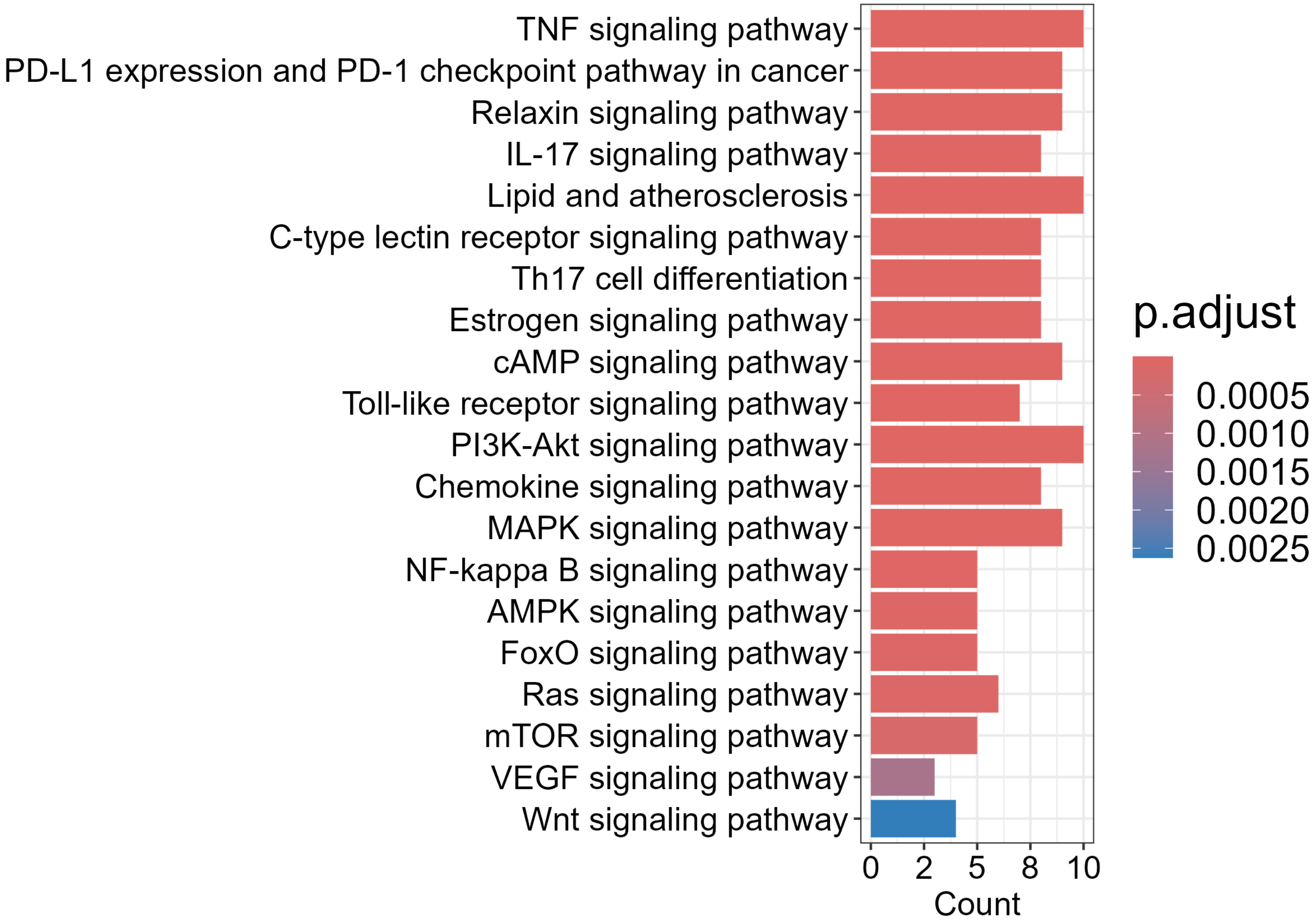

Figure S2. Survival analysis results using CPTAC data. (A) P-values for the sex*score interaction terms in the Cox PH model using upstream signaling proteins and CPTAC data. Only GO terms with significant interaction terms are displayed. (B) Adjusted survival curves for 2 representative GO terms.

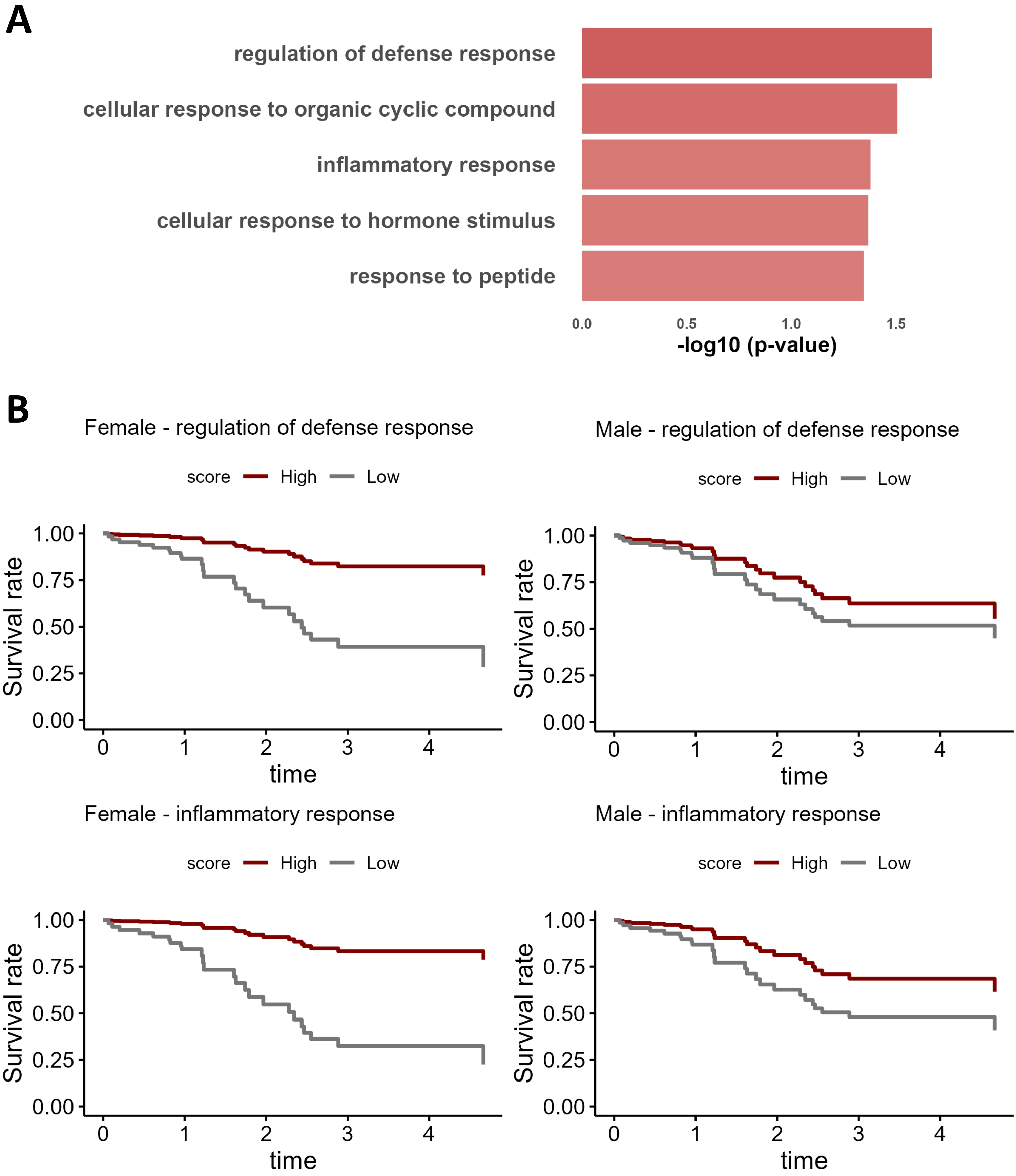

Figure S3. (**A**) Survival analysis using TCGA data reconfirms that sex biases in the impact of immune-related GO terms on patient survival. P-values for the sex*score interaction terms in the Cox PH model are shown, with only significant GO terms displayed. (**B**) Survival analysis using TCGA-LUAD immune deconvolution data from TIMER2.0 elucidates the certain immune cell types associated with sex-biased survival outcomes.

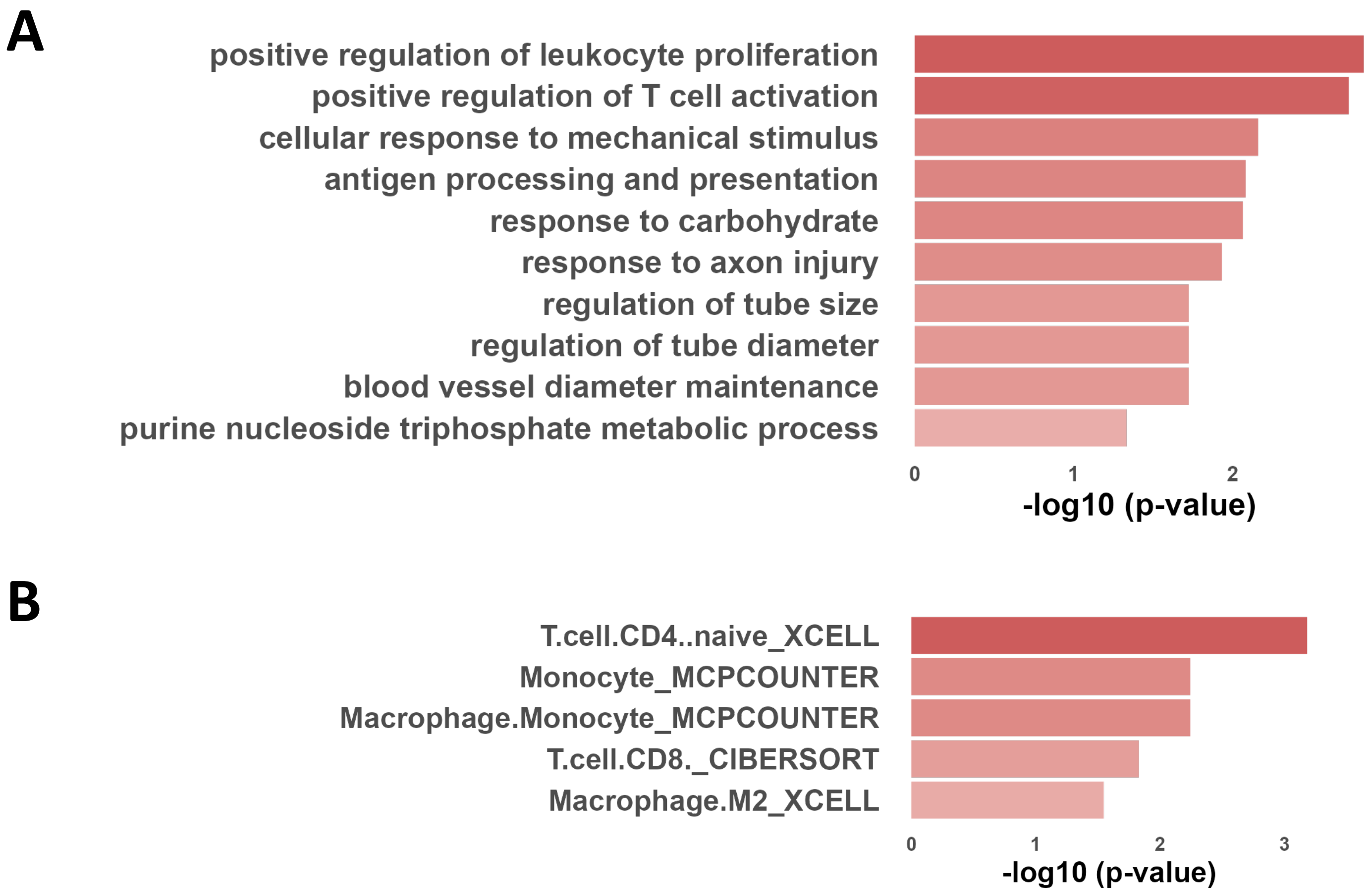

Figure S4. Sex-specific node degrees of HDACs in histone acetylation regulation. Each dot represents an HDAC within the histone acetylation network. Wilcoxon Signed Rank Test.

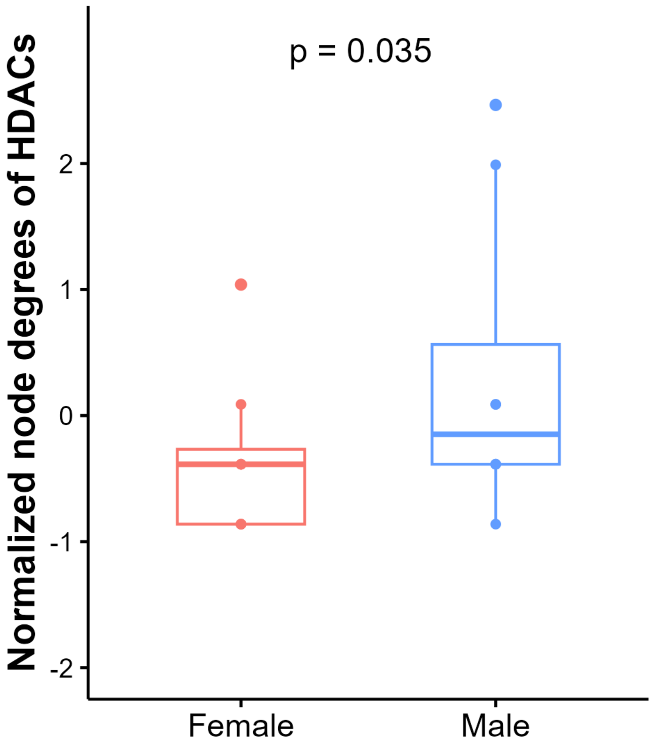

Figure S5. Sex-specific drug effects of seven extra glucocorticoid receptor (NR3C1) agonists. Wilcoxon Rank Sum Test. The p-values were not adjusted.

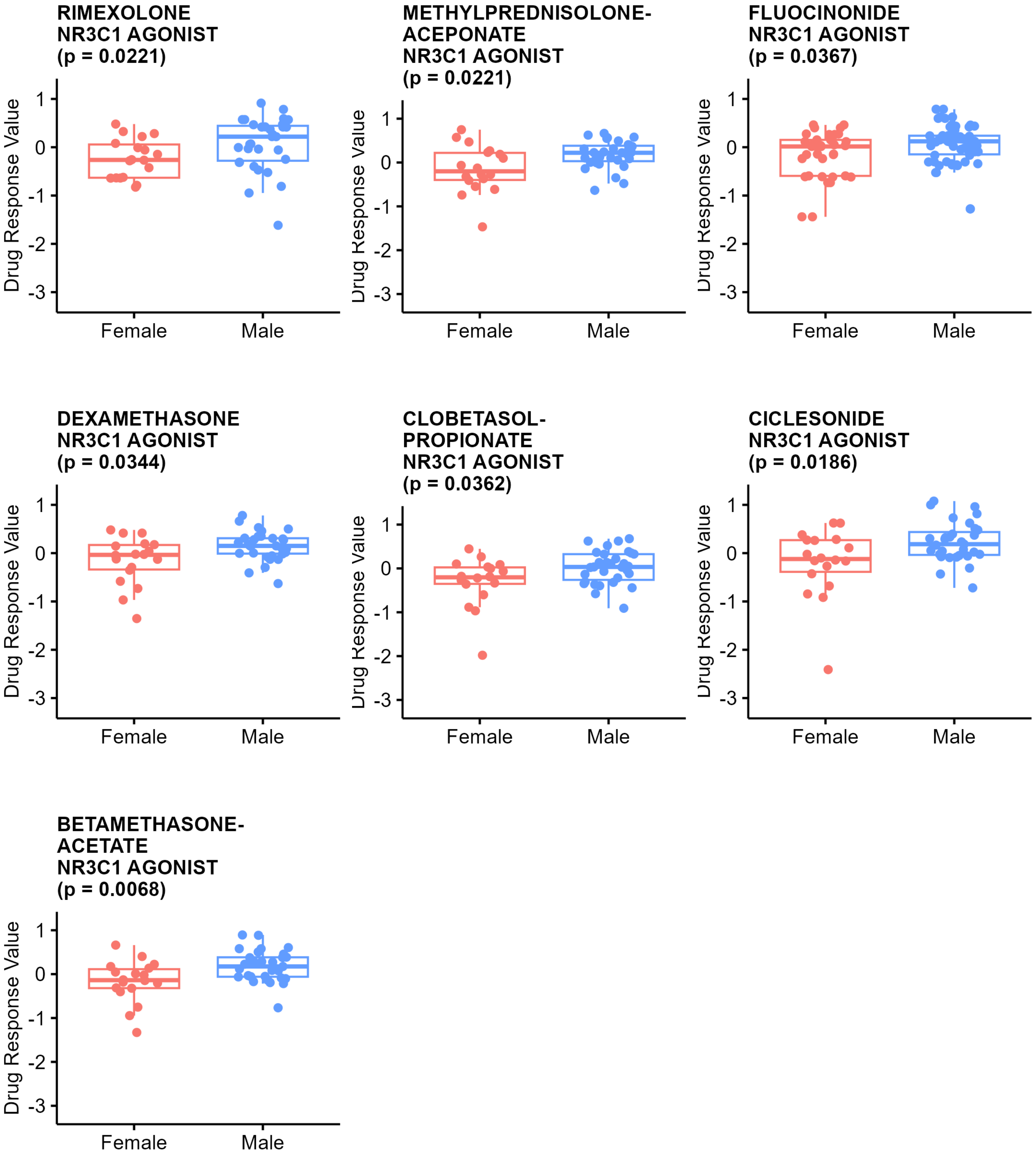

Figure S6. Sex-specific drug effects targeting sex hormone receptors – ESR1 and AR. Wilcoxon Rank Sum Test. The p-values were not adjusted.

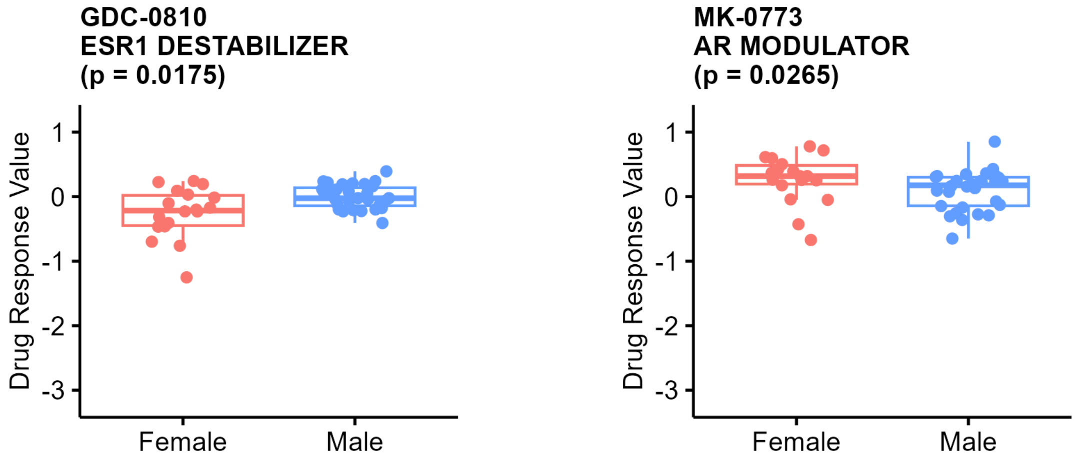

Figure S7. Sex-specific drug effects of MAPK inhibitor. Wilcoxon Rank Sum Test. The p-values were not adjusted.

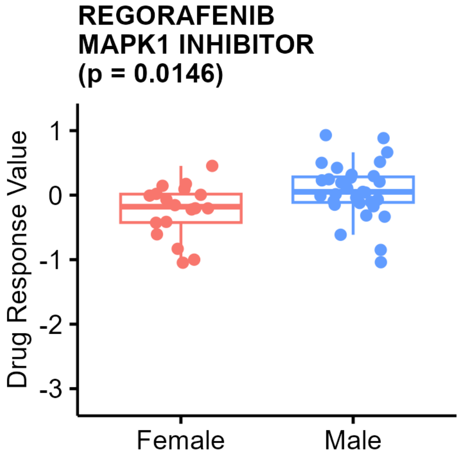

Figure S8. Adjusted p-values of limma differential expression analysis on key nodes.

Orange color stands for the adjusted p-value of limma differential expression analysis. Grey color means the data is missing.

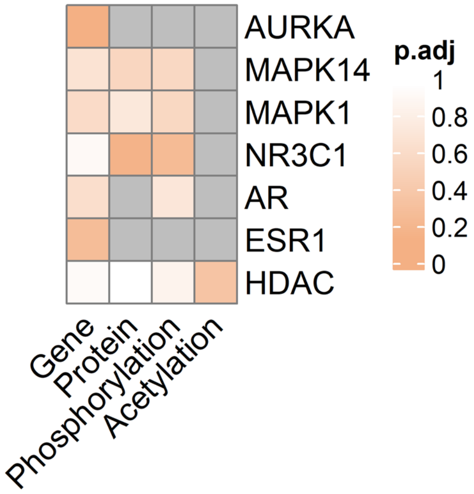

Fig S9. consistency check of validation dataset

A. Scatter plots illustrating the consistency of differential analyses between CPTAC and APOLLO. Limma moderated t-statistic comparing female and male patients.

B. Scatter plots illustrating the consistency of differential analyses between TCGA and GSE68465. Limma moderated t-statistic comparing female and male patients.

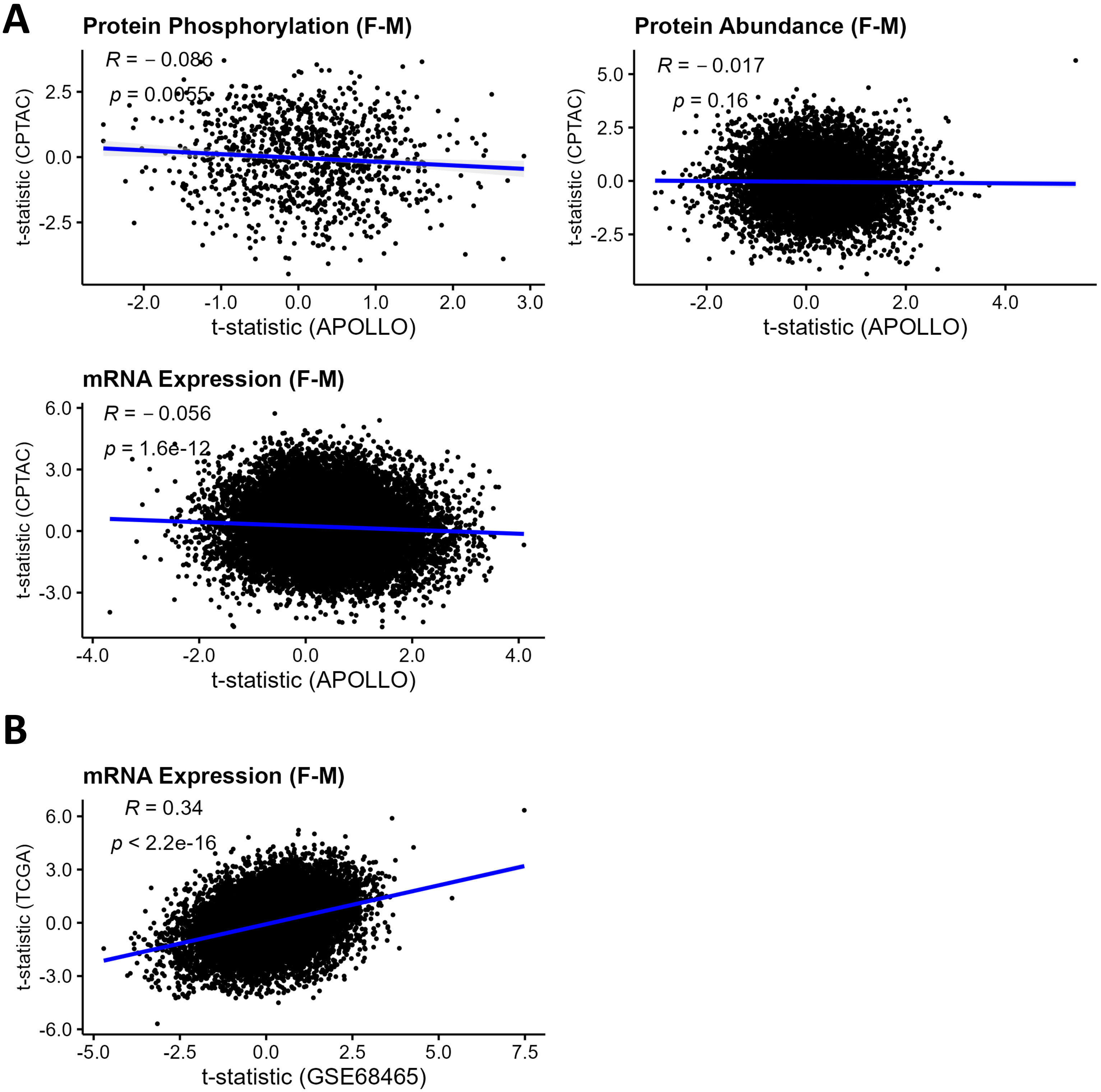

Fig S10: Reproducibility of key nodes (AURKA, AR, NR3C1) using independent datasets.
Scatter plots depicting the consistency of differential kinase and TF activities between discovery (CPTAC, TCGA) and validation (APOLLO, GSE68465) datasets. The left panel shows the limma moderated t-statistic of kinase activities, and the right panel shows the limma moderated t-statistic of TF activities, comparing females with males. Spearman correlation and p-values are included. AURKA, NR3C1, and AR are labelled.

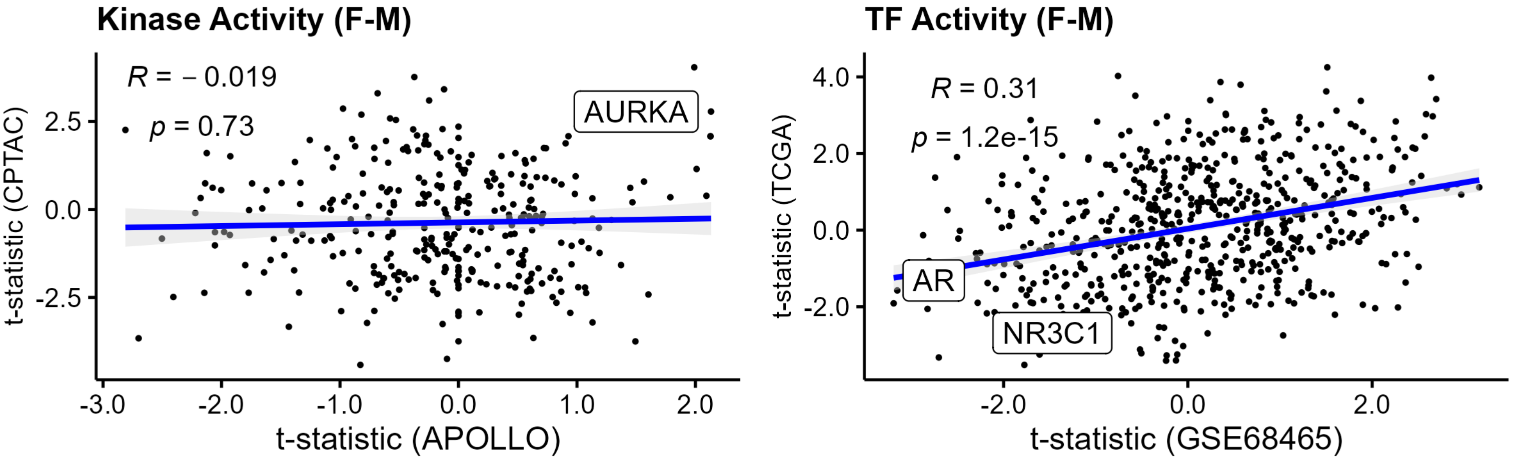

Supplement: Supplementary file 2 — Supplementary Material 2. [file 13293_2025_752_MOESM2_ESM.pdf]
